# The predictive value of a multivariable model based on vaginal *Lactobacillus* relative abundance and microecological features at 24 weeks gestation and before delivery in spontaneous preterm birth: A Prospective cohort study

**DOI:** 10.1371/journal.pone.0339775

**Published:** 2026-02-10

**Authors:** SiLing Ren, Qingrong Wu, TingTing Li, Lingling Jiang, XueJuan Xiao

**Affiliations:** 1 Department of Obstetrics, Chongqing Fuling District Maternal and Child Health Hospital. Fuling District, Chongqing, China; 2 Department of Obstetrics and Gynecology, Chongqing University Fuling Hospital. Fuling District, Chongqing, China; 3 Department of Laboratory Medicine and Blood Transfusion, Chongqing Fuling District Maternal and Child Health Hospital. Fuling District, Chongqing, China; 4 Department of Obstetrics, Chongqing Rongchang District Maternal and Child Health Hospital.Rongchang District, Chongqing, China; Universita degli Studi di Bologna Scuola di Medicina e Chirurgia, ITALY

## Abstract

**Background:**

Vaginal microecological imbalance, particularly reduced *Lactobacillus* dominance, is a significant risk factor for spontaneous preterm birth (sPTB).However, the predictive value of microbial composition, pathogen colonization, and vaginal inflammation biomarkers at different pregnancy stages remains unclear. This study aims to evaluate the predictive potential of vaginal microecology at 24 weeks of gestation and before delivery for spontaneous preterm birth.

**Methods:**

This was a prospective multicenter cohort study. Vaginal swabs were collected at 24 weeks of gestation and before delivery to assess *Lactobacillus* relative abundance(LRA), pathogen colonization, and biochemical markers (pH, hydrogen peroxide, leukocyte esterase, and sialidase). Multivariable logistic regression models, ROC curves, and Kaplan-Meier survival analysis were used to identify predictors of spontaneous preterm birth.

**Results:**

At 24 weeks of gestation, the preterm group showed significantly lower *Lactobacillus* relative abundance, higher AV and Nugent scores, and higher vaginal pH. Logistic regression revealed that an increase in *Lactobacillus* relative abundance(LRA) was negatively associated with sPTB (OR = 0.97, 95% CI = 0.96–0.98, P < 0.001), while higher parity (OR = 2.28, 95% CI = 1.56–3.32, P < 0.001) and a history of late miscarriage/preterm birth (OR = 6.96, 95% CI = 2.77–17.47, P < 0.001) increased the risk. *Lactobacillus* relative abundance(LRA) below 57.5% was linked to increased preterm birth risk in univariate analysis, but this was no longer significant after multivariable adjustment. Parity, late miscarriage/preterm birth history, vaginal pH > 4.5, and GBS positivity remained independent risk factors (AUC = 0.775). Kaplan-Meier survival analysis showed that women with LRA > 72.5% at 24 weeks and LRA > 57.5% before delivery had significantly higher term delivery probabilities (log-rank P < 0.001).

**Conclusion:**

At 24 weeks, reduced vaginal LRA is an independent predictor of spontaneous preterm birth. Early vaginal microbiome assessment, combined with clinical features, can help identify high-risk pregnant women and improve outcomes.

## Introduction

In 2020, global preterm birth rates varied significantly across regions, with incidences ranging from 4% to 16% [1–2]. Complications arising from preterm births became the primary cause of mortality in children under five [3]. In preterm birth cases, some can be attributed to maternal or fetal pathologies that require medical intervention, such as preeclampsia or intrauterine growth restriction, while a significant portion occurs as spontaneous preterm birth (sPTB) [4]. Spontaneous preterm birth (sPTB) was defined as delivery between 28 and 36^+6^ weeks of gestation due to spontaneous uterine contractions leading to cervical dilation or premature rupture of membranes, without medical intervention for maternal or fetal indications (e.g., hypertensive disorders, placental abruption, or fetal distress),accounting for more than 70% of all preterm births. Its occurrence is associated with factors such as infections, cervical insufficiency, and multiple pregnancies [5]. Vaginal microecological imbalance is an important contributor to maternal infections; infections related to this imbalance can easily lead to ascending genital tract infections and premature rupture of membranes, which in turn trigger uterine contractions and ultimately result in spontaneous preterm birth [5–6].

The vaginal microbiota is a complex microbial ecosystem residing in the lower female reproductive tract, predominantly dominated by *Lactobacillus* species, which help maintain the vaginal pH between 3.8 and 4.5, thus creating an acidic protective barrier that inhibits pathogen colonization [7]. In a healthy state, *Lactobacillus* constitutes 50–80% of the vaginal microbiota, and its metabolic products, including lactic acid and hydrogen peroxide, exhibit significant antimicrobial and immunomodulatory effects [8–9]. However, when *Lactobacillus* abundance decreases, and anaerobic bacteria such as *Gardnerella*, *Prevotella*, and *Atopobium* proliferate excessively, the vaginal microecological balance is disrupted, leading to an increase in local inflammatory responses. This imbalance can result in premature rupture of membranes and early uterine contractions, ultimately triggering preterm birth [10].Studies have further highlighted that the composition of the vaginal microbiota undergoes dynamic changes during pregnancy, and these fluctuations are closely associated with the risk of preterm birth [11–13]. In early pregnancy, if *Lactobacillus iners* remains the dominant species, the risk of recurrent preterm birth significantly increases. In contrast, a low-diversity community dominated by *Lactobacillus* in late pregnancy helps to maintain pregnancy stability [14–15].

Therefore, elucidating the relationship between changes in the vaginal microecology at different stages of pregnancy and spontaneous preterm birth is of great significance for understanding the mechanisms of spontaneous preterm birth and developing predictive models. From a morphological perspective, our analysis thoroughly characterized the microbial community in terms of aerobic vaginitis score(AV score), Nugent score, vaginal biochemical markers, and potential pathogenic presence, with particular emphasis on the *Lactobacilli* Relative Abundance(LRA) [11–13]. Functionally, we focused on key indicators pH, hydrogen peroxide,sialidase,and leukocyte esterase.

## Materials and methods

This study was a multicenter prospective cohort study, in accordance with the principles of the Declaration of Helsinki.The study protocol was approved by the Ethics Committees of Chongqing Fuling Maternal and Child Health Hospital (Approval No. FLFY2022032501), Chongqing University Fuling Hospital (Approval No. 2022CDFSFLYYEC-109), and Chongqing Rongchang District Maternal and Child Health Hospital (Approval No. 2022, Ethical Approval, 10th) and data were collected from July 1, 2022, to January 31, 2025. The participants were singleton pregnant women who received prenatal care between July 2022 and January 2025 at Chongqing Fuling District Maternal and Child Health Hospital, Chongqing University Fuling Hospital, and Chongqing Rongchang District Maternal and Child Health Hospital. All participants underwent prenatal care at their respective hospitals. During early pregnancy, obstetricians from the three hospitals approached and recruited eligible women. Those who agreed to participate were registered as potential subjects, and all records were completed by the obstetricians. All data were subsequently centralized, compiled, and analyzed at Chongqing Fuling Maternal and Child Health Hospital during the study period.All participants provided written informed consent prior to inclusion.

The sample size was estimated using Cochran’s formula (n = Z^2^ × p × (1 − p)/ d^2^) [16]. Based on the historical incidence of preterm birth from the three hospitals (9%–15%), an expected preterm birth rate (p) of 12% was assumed, with a 95% confidence level (Z = 1.96) and a 3% margin of error. The minimum calculated sample size was 452, and considering approximately 5% loss to follow-up, the final target sample size was 474.

Gestational age was confirmed by ultrasound performed at 11–14 weeks of gestation using crown–rump length (CRL) to estimate the expected date of delivery [17]. At 24 weeks, baseline maternal characteristics were collected through survey questionnaires, including age, inter-pregnancy interval, pre-pregnancy body mass index (BMI), gravida (G), parity (P), history of cervical surgery, and history of late miscarriage or preterm birth. Inclusion criteria included confirmed singleton pregnancy and no medical and surgical conditions or obstetric complications, such as hypertensive disorders, diabetes, thyroid disease, heart disease, asthma, anemia, or depression. Exclusion criteria included planned cesarean section, use of vaginal or oral antibiotics within the past three months, multiple pregnancies, uterine anomalies, cervical insufficiency, gestational hypertension, heart disease, and gestational diabetes.

At 24 weeks of gestation, participants underwent an initial vaginal microecological assessment. GBS screening of the vagina and rectum was performed between 35 and 37 weeks. If a participant experienced preterm labor before 35 weeks and had not undergone routine GBS screening, the doctor would perform immediate screening upon admission.In the case of symptoms suggestive of preterm labor or the onset of delivery, such as uterine contractions, vaginal bleeding with mucus, or premature rupture of membranes (PROM), participants were advised to avoid sexual intercourse, bathing, and vaginal douching.Pregnant women showing signs of labor at admission had venous blood drawn for CRP measurement, and their vaginal microecological was re-evaluated.Vaginal secretions were collected by obstetricians with more than three years of clinical experience, following strict aseptic protocols in a dedicated examination room,the procedure involved using sterile speculums, sterile gloves, and disposable sterile swabs. Before delivery, samples were collected during the first vaginal examination after hospital admission. After speculum insertion, secretions from the upper third of the vaginal wall were gently removed using a sterile cotton swab. A 10-second rotational sampling was then performed with a sterile swab (Kangjie, Jiangsu Kangjie Medical Equipment Co., Ltd.) in the same region. Samples were immediately stored at 2–8°C and promptly transported to the laboratory for testing.

Samples were diluted to approximately 25–30 cells per high-power field, air-dried, and fixed for Gram staining using the WST-R automatic staining system (Wei Sheng Tai-Ran se, Shandong Xidas Biotechnology Co., Ltd.). At least 50 random high-power fields were examined under an oil immersion lens, and representative images were recorded. The automatic imaging system captured all 50 fields, which were then manually reviewed. If no bacteria of a specific type were observed across all 50 fields, the result was considered negative; detection of any bacteria in at least one field was recorded as positive.

Biochemical parameters including pH, hydrogen peroxide (H₂O₂), leukocyte esterase (LE), and sialidase (SNa) were tested using the Kangjie Vaginal Microecology Diagnostic Kit (KJ2020-VM, four-color test card). The test kit was a dry-chemical multi-analyte detection card containing four independent reaction wells, each designed to detect one of the following parameters: pH, hydrogen peroxide (H₂O₂), leukocyte esterase (LE), and sialidase (SNa). During testing, vaginal swab samples were diluted with normal saline, and an appropriate amount of the suspension was added into each of the four reaction wells. After the reaction was complete, results were interpreted according to the manufacturer’s color chart: light green indicated pH < 4.4, while dark green or blue indicated pH > 4.5; pink or red indicated positive H₂O₂, while colorless indicated negative; blue or dark blue indicated positive LE, while colorless or light yellow indicated negative; and purple indicated positive SNa, while colorless or light yellow indicated negative.

Vaginal microecological evaluation was conducted following the Expert Consensus on the Clinical Application of Vaginal Microecological Evaluation by the Chinese Medical Association [18]. Nugent scoring followed the standard system proposed by Robert P. Nugent (total score 0–10: 0–3 = normal flora, 4–6 = intermediate, and 7–10 = bacterial vaginosis) [19]. Aerobic vaginitis (AV) scoring was based on the criteria established by Dong et al. (total score 0–10: 0–2 = no AV, 3–4 = mild, 5–6 = moderate, and 7–10 = severe) [20]. The relative abundance of *lactobacilli* (LRA) was calculated as the proportion of Gram-positive rods among total bacterial cells on Gram-stained slides [18]. Additional microscopic indicators included leukocytes, clue cells, *Gardnerella*, *Mobiluncus*, Gram-positive cocci, Gram-negative rods, Gram-negative diplococci, yeast spores, yeast hyphae, and *Trichomonas*.

Intervention was provided only to participants diagnosed with abnormal vaginal microecology at 24 weeks of gestation. Treatment protocols were based on the Clinical Practice Guidelines for Bacterial Vaginosis (Chinese Society of Obstetrics and Gynecology, 2016). For bacterial vaginosis (BV), the recommended regimens were oral metronidazole 400 mg twice daily for 7 days or a single 2 g dose, with clindamycin 300 mg twice daily for 7 days as an alternative. For aerobic vaginitis (AV), mild cases were treated with intravaginal clindamycin, while moderate to severe cases received oral cefuroxime 250 mg twice daily for 7 days, with optional concurrent local clindamycin. For vulvovaginal candidiasis (VVC), clotrimazole 0.5 g was administered intravaginally as a single dose or 0.15–0.2 g nightly for 7 consecutive days. For trichomoniasis (TV), oral metronidazole 0.5 g twice daily for 7 days was prescribed.

Pregnant women who met the inclusion criteria were divided into the preterm group (28–36^+6^ weeks) and the term group (≥37 weeks) based on the gestational age at delivery.Pregnancy and delivery management adhered to standardized obstetric protocols to ensure consistency in monitoring and interventions.

### Statistical methods

All statistical analyses were performed using SPSS version 26.0 (IBM Corp., Armonk, NY, USA), and heatmaps and survival curves were generated using Python. Baseline maternal characteristics and vaginal microbiological indicators were analyzed using univariate analysis. Continuous variables were expressed as median (25th–75th percentiles) and compared between groups using the Mann–Whitney U test. Categorical variables were expressed as frequency (percentage) and analyzed using the chi-square test. Variables with P < 0.05 in univariate analysis were included in a multivariable logistic regression model using the stepwise (Wald) method for variable selection. Multicollinearity among variables was assessed using the Variance Inflation Factor (VIF), with VIF values greater than 5 indicating significant multicollinearity. Pearson correlation coefficients among vaginal microbiota indicators were visualized as heatmaps. The predictive performance of vaginal Lactobacillus relative abundance and the logistic regression model was evaluated using receiver operating characteristic (ROC) curve analysis, and the areas under the curves (AUCs) from univariate and multivariable analyses were compared. Survival analysis was performed using Kaplan–Meier curves, and differences in pregnancy duration between groups with different Lactobacillus abundances were assessed with the log-rank test. A two-sided P value < 0.05 was considered statistically significant.

## Results

**Fig 1:**
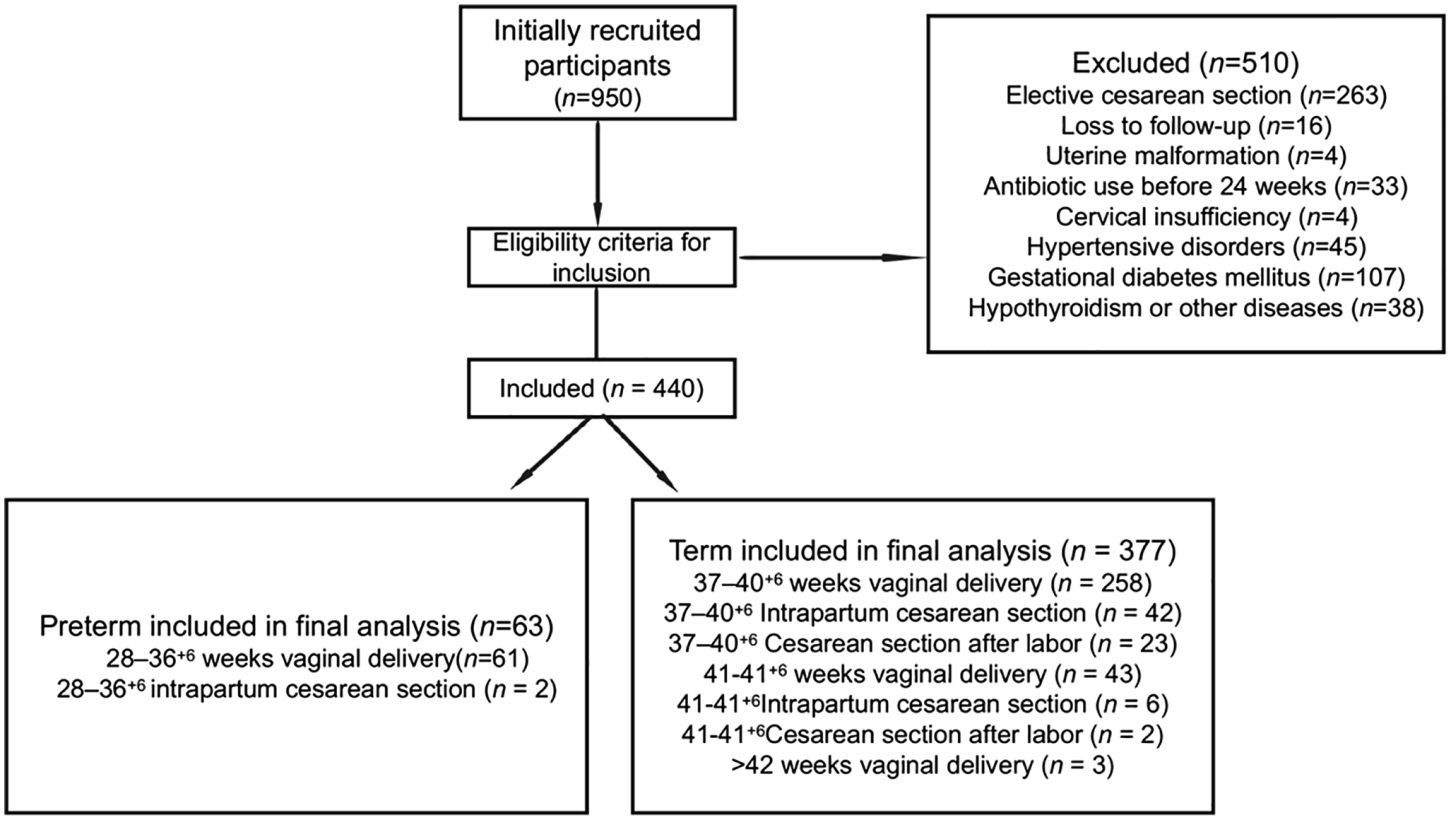
Flow diagram of participant recruitment and inclusion in the study.

A total of 950 pregnant women completed the baseline questionnaire. After excluding those who were lost to follow-up, developed pregnancy complications during follow-up, or underwent medically indicated or planned cesarean deliveries, 440 participants met the inclusion criteria and were included in the final analysis. Participants were divided into preterm and full-term groups based on gestational age. The preterm group (28−36+ ^6^ weeks of gestation) consisted of 63 participants, of whom 61 underwent vaginal delivery, and 2 had a vaginal delivery that was converted to cesarean section (1 due to a previous cesarean section scar and 1 due to fetal distress). The full-term group included 377 participants (≥37 weeks of gestation), with 258 undergoing vaginal delivery between 37 and 40+ ^6^ weeks, 42 requiring a conversion from vaginal delivery to cesarean section (18 due to cephalopelvic disproportion, 12 due to arrested labor, 6 due to fetal distress, and 6 due to amniotic infection), and 23 opting for cesarean section after labor onset (12 due to previous cesarean section, 6 due to macrosomia, and 5 due to pelvic factors). Of the 54 participants delivering at 41−41+ ^6^ weeks, 43 had vaginal deliveries, 6 had a conversion to cesarean section (3 due to cephalopelvic disproportion, 2 due to arrested labor, and 1 due to fetal distress), and 2 chose cesarean section after labor onset (1 due to macrosomia and 1 due to pain intolerance). Three participants delivered after 42 weeks, all by vaginal delivery (Fig 1).

A total of 510 participants were excluded based on the exclusion criteria, 263 participants were excluded due to selective cesarean section, 16 due to loss to follow-up, 4 due to uterine malformations, 33 due to the use of antibiotics before 24 weeks, 4 due to cervical insufficiency, 45 due to hypertensive disorders, 107 due to gestational diabetes, and 38 due to hypothyroidism or other diseases (Fig 1).

**Fig 2:**
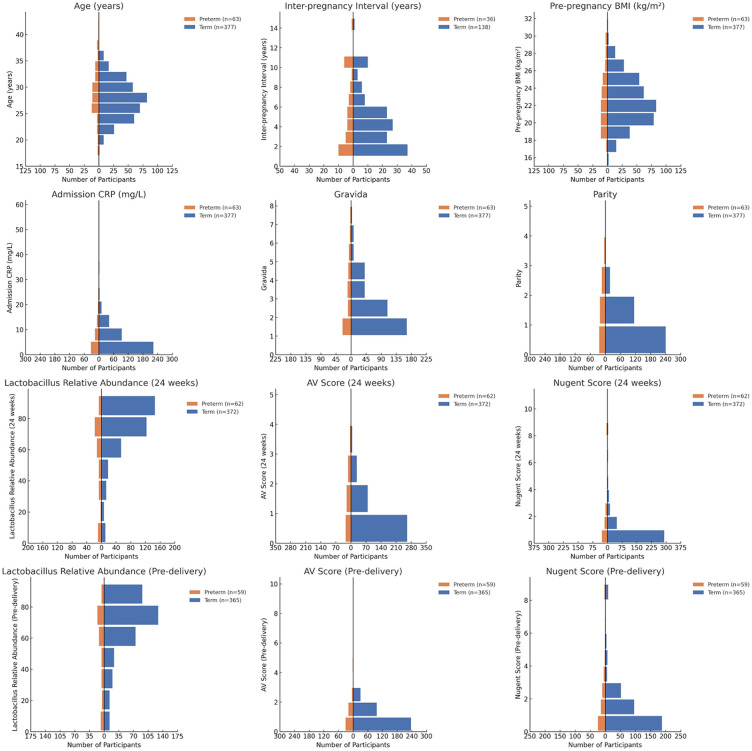
Distribution of Maternal Clinical Characteristics in Term and Preterm Birth Groups.

Univariate analysis revealed no statistically significant differences between the preterm and term groups in terms of age, inter-pregnancy interval, pre-pregnancy BMI, admission CRP, history of cervical surgery (Fig 2). However, significant differences were observed in several variables, including gravida (P = 0.031),parity (P < 0.001), Late Miscarriage/Preterm History (P < 0.001), and *GBS* positivity (P = 0.002) (Table 1).

**Table 1:** Baseline maternal and clinical characteristics of the study cohort stratified by pregnancy outcome.

Characteristics	Total (n = 440)	Term birth (n = 377)	Preterm birth (n = 63)	P-value
Age (years)	28.00 (25.00–31.00)	28.00 (25.00–30.00)	28.00 (26.00–33.00)	0.054
Inter-pregnancy Interval (years)	4.00 (2.00–6.00)	4.00 (2.00–6.00)	4.00 (2.00–8.5)	0.316
Pre-pregnancy BMI (kg/m²)	22.05 (20.50–24.50)	22.00(20.50–24.50)	22.30 (20.50–25.35)	0.700
Admission CRP (mg/L)	3.45 (1.28–10.35)	3.20 (1.275–10.125)	5.70 (1.225–13.575)	0.053
Gravida	3 (2–4)	3 (2–4)	4 (3–5)	0.031
Parity	1 (1–1)	1 (1–1)	1.5 (1–2)	<0.001
Cervical Surgery				
Yes	12 (2.7%)	10 (2.65%)	2 (3.17%)	0.532
No	428 (97.3%)	367(97.35%)	61(96.83%)	
Late Miscarriage/Preterm History				
Yes	26 (5.9%)	15 (3.98%)	11 (17.46%)	<0.001
No	414 (94.1%)	362(96.02%)	52(82.54%)	
GBS				
Positive	32 (7.3%)	21 (5.57%)	11 (17.46%)	0.002
Negative	408 (92.7%)	356 (94.43%)	52 (82.54%)	

Note: No missing data.Age was defined as the woman’s age in years at the time of conception. Inter-pregnancy interval referred to the time (in years) between the previous and the current pregnancy, calculated from the date of the last delivery to the conception date of the current pregnancy. Pre-pregnancy BMI (kg/m^2^) was calculated based on self-reported pre-pregnancy weight and height as weight (kg) divided by the square of height (m^2^). Admission CRP (mg/L) represented the serum C-reactive protein level measured at hospital admission prior to delivery. Gravida denoted the total number of pregnancies, including the current one, regardless of outcome (live birth, miscarriage, or stillbirth). Parity was defined as the number of previous deliveries at or beyond 28 weeks of gestation, regardless of neonatal survival. Cervical surgery referred to any previous cervical procedure that could shorten or weaken the cervix, including cold-knife conization, loop electrosurgical excision procedure (LEEP), laser vaporization, or cryotherapy. Late miscarriage/preterm birth history was defined as a previous spontaneous pregnancy loss between 14 and 27⁺⁶ weeks of gestation or a spontaneous preterm delivery between 28 and 36⁺⁶ weeks. Nulliparous women were defined as those without any previous delivery at ≥28 weeks of gestation. GBS status referred to Group B Streptococcus colonization detected by vaginal and rectal swab cultures performed at 35–37 weeks of gestation.

At 24 weeks’ gestation, significant differences in vaginal microbiota composition were observed between the preterm and term groups. The median relative abundance of *Lactobacilli* was significantly lower in the preterm group than in the term group (P < 0.001). In contrast, the preterm group exhibited higher AV scores (P < 0.001) and Nugent scores (P < 0.001) (Table 2). Among categorical indicators, the preterm group had significantly higher rates of *G+ cocci* positivity (P < 0.001), *Gardnerella* positivity (P = 0.028), *Trichomonas* positivity (P = 0.009), vaginal pH > 4.5 (P < 0.001), sialidase positivity (P = 0.012), and leukocyte esterase positivity (P = 0.007). In contrast, the preterm group had a significantly higher incidence of hydrogen peroxide-negative samples (P = 0.002) (Table 2).

**Table 2:** Univariate analysis of vaginal microecological characteristics between term and preterm birth groups at 24 weeks and before delivery.

	24 Weeks of Gestation		P-Value	Before Delivery		P-Value
	Term birth (n = 372)	Preterm birth (n = 62)		Term birth (n = 365)	Preterm birth (n = 59)	
Lactobacilli Relative Abundance	77.00 (65.00–90.00)	60.00 (33.75–71.25)	<0.001	70.00 (55.00–82.50)	60.00 (30.00–75.00)	<0.001
AV Score	0.00 (0.00–1.00)	1.00 (0.00–2.00)	<0.001	0.00 (0.00–1.00)	0.00 (0.00–1.00)	0.042
Nugent Score	0.00 (0.00–0.00)	1.00 (0.00–2.00)	<0.001	0.00 (0.00–1.00)	1.00 (0.00–2.00)	0.034
leukocytes (Positive)	333(89.50%)	52(83.90%)	0.194	338(92.60%)	54(91.50%)	0.772
Clue Cells (Positive)	3(0.80%)	1(1.60%)	0.461	5(1.40%)	1(1.70%)	0.595
Gardnerella (Positive)	13(3.50%)	6(9.70%)	0.028	21(5.80%)	4(6.80%)	0.756
G+ Cocci (Positive)	0(0.00%)	2(3.20%)	<0.001	1(0.30%)	3(5.10%)	<0.001
G- rods (Positive)	1(0.30%)	0(0.00%)	0.683	0(0.00%)	0(0.00%)	NA
Mobiluncus (Positive)	1(0.30%)	0(0.00%)	0.683	0(0.00%)	0(0.00%)	N/A
Yeast Spores (Positive)	43(11.60%)	9(14.50%)	0.507	24(6.60%)	16(10.20%)	0.318
Yeast Hyphae (Positive)	16(4.30%)	4(6.50%)	0.455	16(4.40%)	4(6.80%)	0.421
Trichomonas (Positive)	1(0.30%)	2(3.20%)	0.009	0(0.00%)	0(0.00%)	N/A
G- Diplococci (Positive)	0(0.00%)	0(0.00%)	NA	0(0.00%)	0(0.00%)	N/A
pH > 4.5	33(8.90%)	16(25.80%)	<0.001	78(21.40%)	29(49.20%)	<0.001
Hydrogen Peroxide (negative)	250(67.20%)	53(86.90%)	0.002	259(71.20%)	54(85.71%)	0.104
Leukocyte Esterase (Positive)	248(66.70%)	52(83.90%)	0.007	238(65.40%)	41(69.50%)	0.537
Sialidase (Positive)	8(2.20%)	5(8.10%)	0.012	15(4.10%)	3(5.10%)	0.734

Note: The total participants included in the analysis were 377 in the term birth group and 63 in the preterm birth group at 24 weeks of gestation. After excluding cases with missing data, the final sample size for the 24-week analysis was 372 in the term birth group and 62 in the preterm birth group.

Similar trends persisted immediately before delivery. The median relative abundance of *Lactobacilli* remained significantly lower in the preterm group than in the term group (P < 0.001). The preterm group continued to exhibit significantly higher AV scores (P = 0.042) and Nugent scores (P = 0.034). Additionally, the preterm group had higher rates of *G+ cocci* positivity (P < 0.001) and pH > 4.5 (P < 0.001). No other significant differences were observed (Table 2).

**Fig 3:**
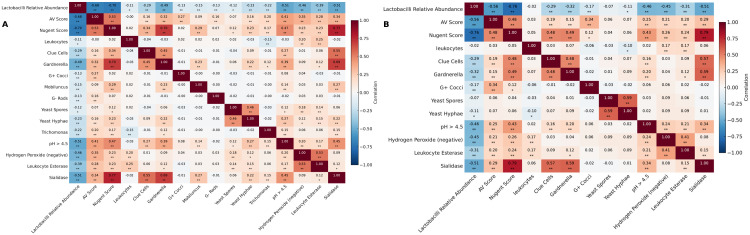
Correlation heatmaps of vaginal microecological indicators and Lactobacillus relative abundance at 24 weeks and before delivery. **A.** Correlation Heatmaps of Vaginal Microecological Indicators at 24 Weeks of Gestation. **B.** Correlation Heatmaps of Vaginal Microecological Indicators Before Delivery. Notes:* Correlation is significant at the 0.05 level (two-tailed). ** Correlation is significant at the 0.01 level (two-tailed).

The collinearity analysis of vaginal microecological indicators showed, at 24 weeks of gestation, the VIF value of the Nugent score was 4.619, followed by sialidase (VIF = 3.835) and *Lactobacillus* relative abundance (VIF = 3.485) (Table 3).Pearson correlation analysis (Fig 3A) further demonstrated that at 24 weeks, *Lactobacillus* relative abundance was significantly negatively correlated with the AV score (r = −0.68), Nugent score (r = −0.70), leukocytes (r = −0.11), Clue cells (r = −0.29), *Gardnerella* (r = −0.49), *G+ cocci* (r = −0.13), Yeast spores (r = −0.12), Yeast hyphae (r = −0.23), and *Trichomonas* (r = −0.15).In addition, *Lactobacillus* relative abundance (LRA) was significantly negatively correlated with pH > 4.5 (r = −0.51), lydrogen peroxide negative status (r = −0.46), leukocyte esterase (r = −0.39), and sialidase (r = −0.51).Positive correlations were observed between Yeast hyphae and Yeast spores (r = 0.46), between *Gardnerella* and Clue cells (r = 0.45), and between lydrogen peroxide negative status and leukocyte esterase (r = 0.53).

**Table 3:** Collinearity Analysis of Clinical and Vaginal Microecological Variables at 24 Weeks and Pre-Delivery.

variable	24-week		Pre-delivery	
	Tolerance	VIF	Tolerance	VIF
GBS	0.935	1.069	0.945	1.059
Gravida	0.468	2.136	0.47	2.127
Parity	0.442	2.262	0.464	2.154
Late Miscarriage/Preterm History	0.960	1.041	0.941	1.063
Lactobacilli Relative Abundance	0.287	3.485	0.323	3.093
AV Score	0.465	2.149	0.66	1.514
Nugent Score	0.216	4.619	0.266	3.754
leukocytes	0.865	1.156	0.901	1.109
Clue Cells	0.596	1.678	0.63	1.587
Gardnerella	0.375	2.664	0.6	1.667
G+ Cocci	0.865	1.157	0.836	1.196
Mobiluncus	0.852	1.174	N/A	N/A
G- Rods	0.945	1.059	N/A	N/A
Yeast Spores	0.751	1.331	0.642	1.557
Yeast Hyphae	0.700	1.429	0.625	1.601
Trichomonas	0.853	1.172	N/A	N/A
G- Diplococci	N/A	N/A	N/A	N/A
pH > 4.5	0.646	1.548	0.78	1.282
Hydrogen Peroxide (negative)	0.599	1.67	0.736	1.358
Leukocyte Esterase	0.660	1.516	0.787	1.271
Sialidase	0.261	3.835	0.273	3.666

Before delivery, the overall VIF values decreased, with the Nugent score (VIF = 3.754), sialidase (VIF = 3.666), and *Lactobacillus* relative abundance (VIF = 3.093) showing the largest values, while all other variables had VIF values below 3 (Table 3).Pearson correlation analysis (Fig 3B) showed that *Lactobacillus* relative abundance was significantly negatively correlated with the AV score (r = −0.56) and Nugent score (r = −0.76), and negatively correlated with Clue cells (r = −0.29), *Gardnerella* (r = −0.32), *G+ cocci* (r = −0.17), and Yeast hyphae (r = −0.11).It was also negatively correlated with pH > 4.5 (r = −0.46), hydrogen peroxide negative status (r = −0.45), leukocyte esterase (r = −0.31), and sialidase (r = −0.51).Regarding pathogen interactions, Yeast hyphae were positively correlated with Yeast spores (r = 0.59), *Gardnerella* was positively correlated with Clue cells (r = 0.48), and hydrogen peroxide negative status was positively correlated with leukocyte esterase (r = 0.41).

The stepwise (Wald) multivariable logistic regression analyses conducted at 24 weeks of gestation and before delivery included all univariate variables significantly associated with spontaneous preterm birth, including both vaginal microecological characteristics and baseline patient features. These analyses revealed that an increase in *Lactobacilli* Relative Abundance (LRA) at 24 weeks was independently associated with a decreased risk of spontaneous preterm birth (P < 0.001, OR = 0.974, 95% CI: 0.963–0.985). In contrast, factors such as parity (P < 0.001, OR = 2.276, 95% CI: 1.562–3.315) and a positive history of late miscarriage/preterm birth (P < 0.001, OR = 6.959, 95% CI: 2.773–17.465) were independently associated with an increased risk of spontaneous preterm birth.Before delivery, the final model included four variables that were significant independent risk factors for spontaneous preterm birth: Parity (P < 0.001, OR = 2.642, 95% CI: 1.800–3.877), a positive Late Miscarriage/Preterm History (P < 0.001, OR = 5.601, 95% CI: 2.204–14.230), Pre-delivery Vaginal pH > 4.5 (P < 0.001, OR = 3.171, 95% CI: 1.704–5.902), and *GBS* Positivity (P = 0.020, OR = 2.969, 95% CI: 1.183–7.452), all of which were independently associated with an increased risk of spontaneous preterm birth (Table 4).

**Table 4:** Final multivariable logistic regression models for predictors of spontaneous preterm birth (sPTB) at different time points.

Variable	B (Estimation)	Standard Error	OR (Exp(B))	P value	95% CI	Model Timepoint
Late Miscarriage/ Preterm History	1.94	0.469	6.96	<0.001	2.77–17.47	24 weeks
Parity	0.822	0.192	2.28	<0.001	1.56–3.32	24 weeks
Lactobacillus Relative Abundance	−0.027	0.006	0.97	<0.001	0.96–0.98	24 weeks
Late Miscarriage/ Preterm History	1.723	0.476	5.60	<0.001	2.20–14.23	Before delivery
Parity	0.971	0.196	2.64	<0.001	1.80–3.88	Before delivery
GBS (Positive)	1.088	0.469	2.97	0.020	1.18–7.45	Before delivery
pH > 4.5	1.154	0.317	3.17	<0.001	1.70–5.90	Before delivery

Note: At 24 weeks of gestation, 6 cases with missing data were excluded, resulting in a final sample size of 434 participants. For the pre-delivery analysis, 16 cases with missing data were excluded, yielding a final sample size of 424 participants.All missing values were handled using the listwise deletion method.

**Fig 4:**
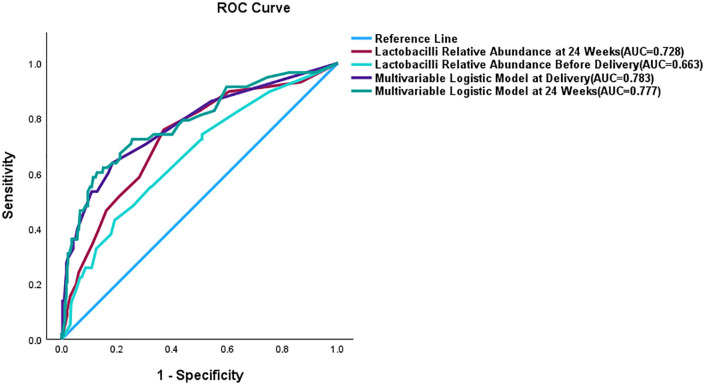
ROC Curve Comparison of *Lactobacilli* Relative Abundance and Multivariable Logistic Models for Predicting Preterm Birth.

Receiver operating characteristic (ROC) curve analysis was performed to evaluate the predictive performance of both the single-variable indicator (*Lactobacilli* Relative Abundance, LRA) and the multivariable logistic regression models.When LRA was analyzed as a single predictor, the results showed that at 24 weeks of gestation, the optimal cutoff value for predicting spontaneous preterm birth (sPTB) was 72.5%, with an area under the curve (AUC) of 0.728. Before delivery, the optimal cutoff value of LRA was 57.5%, with an AUC of 0.663 (Fig 4).

Subsequently, the significant variables identified in the multivariable logistic regression analyses (the 24 weeks model included LRA, Parity, and Late Miscarriage/Preterm; the pre-delivery model included Parity, Late Miscarriage/Preterm, pre-delivery vaginal pH > 4.5, and *GBS* positivity) were incorporated into comprehensive predictive models. The predicted probabilities derived from these models were then used for ROC curve analysis. The results indicated that the multivariable model achieved an AUC of 0.783 at 24 weeks and 0.777 before delivery (Fig 4).

**Fig 5:**
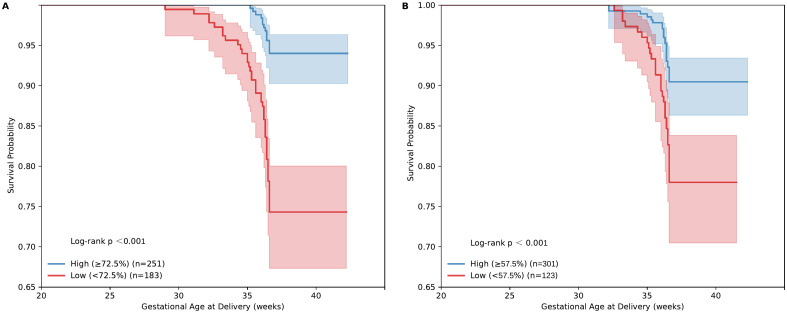
Kaplan–Meier survival curves for term delivery based on vaginal Lactobacilli relative abundance at 24 weeks and before delivery. **A.** Kaplan–Meier Survival Curve for Term Delivery Based on *Lactobacilli* Relative Abundance at 24 Weeks of Gestation. **B.** Kaplan–Meier Survival Curve for Term Delivery Based on *Lactobacilli* Relative Abundance Before Delivery.

Survival analysis further demonstrated that women with higher LRA (≥72.5%) at 24 weeks maintained a significantly greater cumulative probability of term delivery throughout pregnancy (log-rank test, P < 0.001), whereas the curve for the low-LRA group (＜72.5%) declined sharply between 32 and 36 weeks (Fig 5A). In the pre-delivery analysis, women with higher LRA (≥57.5%) similarly exhibited a higher probability of term delivery (log-rank test, P < 0.001) (Fig 5B).

## Discussion

In this prospective cohort study, we comprehensively evaluated the associations between vaginal microecological characteristics and maternal clinical factors at 24 weeks of gestation and before delivery in relation to the risk of spontaneous preterm birth (sPTB). The results showed that at 24 weeks, a higher *Lactobacillus* relative abundance (LRA) was independently associated with a reduced risk of sPTB, whereas a history of late miscarriage or preterm birth and higher parity were independent risk factors. By the time of delivery, although an *Lactobacillus* relative abundance (LRA)above 57.5% remained associated with a lower sPTB risk in univariate analysis, it was no longer an independent predictor in the multivariable logistic regression model. At this stage, late miscarriage/preterm history, higher parity, *GBS* positivity, and elevated vaginal pH (>4.5) were identified as independent risk factors.

Several cohort studies have reported that vaginal microbiota dominated by anaerobic *non-Lactobacillus* species (Community State Type IV, CST IV) is associated with an increased risk of spontaneous preterm birth (sPTB), whereas *Lactobacillus*-dominated communities may exert a protective effect by maintaining a low vaginal pH, producing lactic acid and hydrogen peroxide (H₂O₂), and inhibiting the growth of pathogenic microorganisms [21–22]. The findings of the present study support this protective association. At 24 weeks of gestation, a higher *Lactobacillus* Relative Abundance (LRA) was accompanied by lower Aerobic Vaginitis (AV) score and Nugent score, together with multiple favorable vaginal microecological features [23–24]. By the time of delivery, the magnitude of differences in vaginal microbiota composition and related microecological parameters between the term and preterm groups was markedly reduced, and the association between *Lactobacillus* Relative Abundance (LRA) and sPTB risk was no longer significant in the multivariable model. This attenuation may reflect increased overall variability of the vaginal microecology in late pregnancy, as well as the combined influence of other clinical factors and medical interventions on risk assessment.

Apart from *Lactobacillus* relative abundance (LRA), several other pathogen factors were significantly associated with the risk of spontaneous preterm birth (sPTB) in univariate analysis. At 24 weeks of gestation, the positivity rates of *Gardnerella*, Gram-positive cocci, and *Trichomonas* were significantly higher in the preterm birth group compared to the term birth group, especially the positivity rate of Gram-positive cocci, which showed a particularly significant difference in the preterm group (P < 0.001). Although the data for Gram-positive cocci were sparse (n = 2) and were not retained in the final logistic regression model, the potential role of Gram-positive cocci in the occurrence of spontaneous preterm birth cannot be excluded. Future studies could further validate the robustness of these results by increasing the sample size.

In the univariate analysis at 24 weeks of gestation, pH > 4.5, leukocyte esterase positivity, hydrogen peroxide negativity, and sialidase positivity were all significantly associated with the risk of preterm birth. An elevated vaginal pH promotes the colonization and proliferation of pathogens, disrupts the vaginal barrier, triggers immune responses, and facilitates pathogen co-colonization.Normally, *Lactobacillus* (especially *Lactobacillus crispatus*) maintains a low pH (3.8–4.5) via lactic acid, H₂O₂ and produce immunomodulators (indole-3-lactic acid, SCFAs) that via AhR/GPR receptors suppress NF-κB, reducing pro-inflammatory cytokines (IL-1β, IL-6, TNF-α) [25–26]. When lactic acid,H₂O₂ decline raises pH > 4.5, anaerobes proliferate, forming biofilms that compromise epithelial barriers and increase infection susceptibility.When diminished, this anti-inflammatory protection is lost, triggering chemokine (CXCL8) upregulation and abnormal ECM remodeling via MMP-8 activation – promoting collagen degradation, cervical softening, and preterm birth initiation.Elevated vaginal pH selectively promotes BV-associated anaerobe overgrowth and enhances Gram-positive cocci (e.g., *GBS*) adhesion/biofilm formation on vaginal epithelium [27]. These pathogens secrete hemolysins, hyaluronidases, and phospholipases that degrade fetal membranes and induce COX-2 expression, boosting PGE₂ production to trigger uterine contractions and PROM. BV anaerobes also release LPS/flagellin that activate TLR4-NF-κB pathway, amplifying inflammation and uterine contractions, creating a barrier disruption-inflammation-contraction vicious cycle.

Late miscarriage/preterm history and parity emerged as independent risk factors for spontaneous preterm birth (sPTB) at both 24 weeks of gestation and before delivery in our multivariate analyses. Previous studies have repeatedly demonstrated that a history of late miscarriage or preterm birth is one of the strongest predictors of future preterm delivery, with relative risks as high as 4–6 fold [28–29]. This may be due to prior cervical trauma, membrane weakening, or chronic endometrial inflammation, all of which can alter cervical biomechanical properties and increase the likelihood of premature cervical shortening or membrane rupture. Parity was also positively associated with sPTB risk in our study, with higher parity linked to increased mechanical stress on the cervix, reduced tissue elasticity, and diminished Extracellular Matrix remodeling capacity.

The significant predictive power of *Lactobacillus* Relative Abundance(LRA)at 24 weeks suggests that early vaginal microbiome assessment may be more effective than later assessments in identifying high-risk women for sPTB, supporting its inclusion in clinical practice. Studies show that probiotic supplementation can reduce the recurrence of BV and improve vaginal pH stability [30]. For individuals with *Lactobacillus* depletion, vaginal microbiota transplantation (VMT) could be an effective strategy, but its clinical data for sPTB prevention is limited. Prenatal counseling should emphasize avoiding unnecessary antibiotics and frequent vaginal douching.

This study has several limitations. First, the absence of 16S rRNA sequencing or metagenomic analysis restricted our ability to explore the dynamic changes of the vaginal microbiota and the interaction mechanisms between *Lactobacillus* and specific pathogens in depth. Second, participants who underwent planned cesarean delivery were excluded. Because the timing and mode of delivery in this group are influenced by human decision-making factors, such exclusion may have introduced selection bias, resulting in a study population more representative of spontaneous labor. This could potentially underestimate the differences in vaginal microecology and preterm birth risk associated with other delivery modes. In addition, we excluded patients with gestational diabetes mellitus (GDM) and gestational hypertension (GH), and other related conditions, as these patients may require medical interventions for early termination of pregnancy, which mechanistically differs from spontaneous preterm birth. However, considering that GDM and GH occur in approximately 5–10% of pregnancies, these exclusion criteria may have introduced selection bias, thereby limiting the external validity of our findings to broader populations.

In addition, this study lacked information on several additional potential confounding factors, such as smoking history, a history of sexually transmitted infections (e.g., *Chlamydia trachomatis*), and recent semen exposure. These factors may alter the vaginal microecological environment, affect local immune responses, or promote ascending infections, thereby influencing the assessment of preterm birth risk. The absence of such information prevented adequate adjustment in the analysis and may have resulted in residual confounding bias.Future studies should include larger, multicenter cohorts to validate the threshold of Lactobacillus relative abundance and integrate multi-omics approaches—including metagenomics, transcriptomics, and metabolomics—to elucidate the underlying biological mechanisms. Moreover, randomized controlled trials (RCTs) are needed to evaluate the safety and efficacy of probiotic interventions for the prevention of spontaneous preterm birth.

## Supporting information

S1 Table Participant Questionnaire. (DOCX)
