# Supplementary material for: The predictive value of a multivariable model based on vaginal Lactobacillus relative abundance and microecological features at 24 weeks gestation and before delivery in spontaneous preterm birth: A Prospective cohort study
Source: PLoS One. 2026 Feb 10;21(2):e0339775. doi: 10.1371/journal.pone.0339775 (PMC12890100; doi:10.1371/journal.pone.0339775)
Supplement: S1 Table — (DOCX) [file pone.0339775.s001.docx]

**Participant Questionnaire**

**Name：**

**Study ID ： ________
Hospital ： □ FLMCH □ CUFH □ RCMCH
Date： ____
Gestational age today：__ weeks**

**Section A.**

**A1. Maternal age (years)**

**A2. height**

**A3. Pre-pregnancy weight**

**Section B.**

**B1. Gravida (Number of Pregnancies,including the current pregnancy)**

**B2. Parity（Number of deliveries at or beyond 28 weeks of gestation）**

**B3. Inter-pregnancy interval
Date of Last Delivery: ________ 与 Date of Last Menstrual Period: ________**

**Section C.**

**C1. Have you ever had any cervical procedures?

□ Cold-knife conization
□ LEEP**

**□ Laser vaporization
□ Cryotherapy
□ Other (specify) ：____________**

**Section D.**

**D1. Late miscarriage (14–27⁺⁶ weeks)**Have you ever experienced a pregnancy loss between 14 and 27 weeks of gestation?
□ No  □ Yes (please specify the number of occurrences: [ ])
If “Yes,” please indicate the most likely cause (e.g., infection, premature rupture of membranes, etc.):
[ ____________________________________________________________ ]

**D2. Preterm birth (28–36⁺⁶ weeks)**
Have you ever delivered a baby between 28 and 36 weeks of gestation?
□ No  □ Yes (please specify the number of occurrences: [ ])
If “Yes,” please indicate the most likely cause (e.g., premature rupture of membranes, infection, placental abruption, hypertensive disorder, fetal distress, etc.):
[ ____________________________________________________________ ]

**Section E.**

**E1. Do you have or have you ever been diagnosed with any of the following medical conditions (before or during this pregnancy)?**
(Please tick all that apply.)

□ None / No known medical conditions
□ Hypertension / High blood pressure
□ Diabetes mellitus (type 1 / type 2 / gestational)
□ Thyroid disease (e.g., hypothyroidism, hyperthyroidism)
□ Heart disease (e.g., congenital, rheumatic, arrhythmia)
□ Asthma or other chronic respiratory disease
□ Autoimmune or connective-tissue disorder
□ Chronic kidney disease
□ Anemia or hematologic disorder
□ Depression or other mental-health condition
□ Other (please specify): _________________________________________

**E2. Were any of these conditions newly diagnosed during this pregnancy?**
□ No  □ Yes (please specify which: ________________________________)

**E3. Are you currently taking any medications for these conditions?**
□ No  □ Yes (please list medication name(s): _________________________)
